# Decreased Circulating Red Cell Mass Induced by Intravenous Acepromazine Administration Alters Viscoelastic and Traditional Plasma Coagulation Testing Results in Healthy Horses

**DOI:** 10.3390/ani14213102

**Published:** 2024-10-28

**Authors:** Ina Mersich, Rebecca C. Bishop, Sandra Diaz Yucupicio, Ana D. Nobrega, Scott M. Austin, Anne M. Barger, Meghan E. Fick, Pamela Anne Wilkins

**Affiliations:** 1 Department of Veterinary Clinical Medicine, University of Illinois Urbana-Champaign, 1008 West Hazelwood Drive, Urbana, IL 61802, USA; imersich@illinois.edu (I.M.); anadn@illinois.edu (A.D.N.); smaustin@illinois.edu (S.M.A.); mefick@uga.edu (M.E.F.); 2 Veterinary Clinical Sciences, Washington State University, Ott Rd #110, Pullman, WA 99163, USA; 3 Department of Small Animal Medicine and Surgery, University of Georgia, 501 D. W. Brooks Drive, Athens, GA 30602, USA

**Keywords:** coagulopathy, anemia, hypercoagulability, acepromazine, equine

## Abstract

Coagulopathy is common in equine critical illness, with its early recognition being crucial for patient management and prognosis. In vitro viscoelastic (VE) hypercoagulability with decreased RCM/PCV has been demonstrated in dogs but not horses. Our objective was to evaluate the effects of acepromazine-induced (0.1 mg/kg IV) decreased RCM on VE and plasma coagulation parameters using a prospective interventional study of eight adult horses. Complete blood count (CBC), fibrinogen, prothrombin time (PT), partial thromboplastin time (PTT), packed cell volume (PCV), total solids (TS), and VCM Vet™ VE testing performed at baseline (T0), 1 h (T1), and 12 h (T2) post acepromazine administration. Splenic volume was determined ultrasonographically. The results were analyzed using one-way repeated measures ANOVA with Tukey’s post hoc HSD test to determine the effect of time (sample). PCV decreased 13% points following acepromazine administration from T0 to T1 (*p* < 0.001), remaining decreased at T2 (*p* < 0.001). Splenic volume increased from T0 to T1 (*p* = 0.04) and was not different from baseline at T2. Maximal clot formation (MCF) increased from T0 (*p* = 0.03). PTT decreased from T0 to T1 and increased at T2 (*p* = 0.03). No other coagulation parameters were significantly altered. This study demonstrates a non-inflammatory acute model of anemia in horses that impacts VE and plasma-based testing.

## Simple Summary

Clotting (coagulation) is an essential body function, ensuring blood clots can form and resolve properly when needed. States of increased (hypercoagulability) or decreased (hypocoagulability) clotting are commonly observed in various disease states. Viscoelastic testing presents a method of measuring adequate clotting, ultimately showing a graph depicting the entire clotting process, from clot formation to clot lysis. The advantage of this method over others is that it provides a global view on the coagulation status the patient. Red blood cell (RBC) values have been shown to affect viscoelastic testing; however, it is unclear to date if this represents a true change or an artifact. In this study, anemia (less RBC in blood stream) was medically induced by the administration of acepromazine. This effect was caused by sequestration of RBC in the spleen, which was confirmed via ultrasound examination of the spleen (splnic size increase). The reduction in RBC led to hypercoagulability, shown as an increase in one viscoelastic testing parameter (maximum clot formation) and a decrease in the partial thromboplastin time. This study showed that acepromazine can be used to safely induce anemia in healthy horses. Anemia caused an increase in spleen size and laboratory, but not clinical, hypercoagulability.

## 1. Introduction

Coagulation is an essential function of the body, ensuring a balance between clot formation and lysis [1]. Both hypercoagulability, potentially causing thrombotic events, and hypocoagulability, leading to an increased risk of hemorrhage, can occur in critically ill horses [2]. Several studies have demonstrated that equine patients suffering from severe gastrointestinal diseases such as colitis, enteritis, strangulating intestinal lesions, and intestinal displacement are at risk of developing coagulopathy [3,4]. Recognition of abnormal coagulation becomes essential for prognosis prediction and the development of treatment strategies [2].

Conventional equine coagulation testing panels include prothrombin time (PT), partial thromboplastin time (aPTT), fibrinogen, and antithrombin (AT) [5]. Fibrin degradation products (FDP) serve as a measure of fibrinolysis, and platelet count is important to both hemostasis and clot formation. While each of these tests individually evaluates components of the hemostasis and coagulation processes, several tests are needed for a, still relatively incomplete, evaluation [6]. Viscoelastic (VE) testing has been utilized for coagulation assessment in several species and was first described in 1948 [7]. This method, compared to conventional assays, performs a more global assessment of coagulation, including cellular and plasma-derived components [6]. Since its inception, VE testing has been used in human patients as a transfusion guide, for the assessment of trauma patients, and for intra-operative monitoring during organ transplantation [8,9,10]. In veterinary medicine, VE testing has been reported in dogs with immune-mediated hemolytic anemia, canine parvovirus infection, and in patients with malignant neoplasia [11,12,13,14]. In horses, VE testing has been reported in patients with gastrointestinal disease, monitoring effects of administered heparin, and in the evaluation of the hemostatic system in equine neonates, among others [15,16,17].

Studies in human and veterinary medicine suggest a connection between red blood cell mass (RCM) and hyper- or hypocoagulability, as assessed by VE testing. In patients with altered RCM, changes in VE parameters may be artifactual, presenting a challenge when interpreting results. Alterations in VE parameters have been reported in equine patients with increased circulating RCM [18,19,20]. To our knowledge, there are no studies to date examining the effect(s) of anemia, decreased RCM, on VE testing in horses [20]. Induction of anemia in healthy animals can be achieved by blood removal or administration of medications which alter RCM, measured using packed cell volume (PCV) [19,21]. Acepromazine and other sedatives have been shown to decrease PCV in horses and other species [22,23,24,25,26]. Reduction in red cell parameters and PCV after acepromazine administration has been attributed to splenic sequestration of red blood [21]. Transabdominal ultrasonography has been used to measure splenic volume changes in horses following the administration of phenylephrine and detomidine [20,27].

The primary objective of this study was to determine the effects of decreased RCM on PCV, splenic volume, VE coagulation, and plasma coagulation/hemostatic parameters in healthy horses. We hypothesized that acepromazine-induced decreased RCM would be associated with both increased splenic volume and altered VE coagulation profiles. A secondary objective was to determine any direct effects of acepromazine on VE coagulation when added ex vivo to native whole equine blood; we hypothesized that the direct addition of acepromazine to native whole blood would not change VE coagulation parameters.

## 2. Materials and Methods

### 2.1. Horses

Eight healthy adult horses from the University of Illinois College of Veterinary Medicine research herd were used for this study. There were seven mares and one gelding enrolled in the study. The horses were of multiple breeds, including three Arabian, two Quarter Horses, and one each Standardbred, Warmblood, and Tennessee Walking Horse. The age ranged from 7 to 18 years, and body weight ranged from 431 to 639 kg. Enrolled horses were considered healthy based on normal physical examination, as well as unremarkable complete blood count (CBC), plasma chemistry, and fibrinogen concentration results. Health status was determined no longer than 48 h prior to study enrollment. The study protocol was approved by the Institutional Animal Care and Use Committee (IACUC protocol #22184).

### 2.2. In Vivo Acepromazine-Induced Anemia Model

Horses were manually restrained in stocks, and an intravenous (IV) catheter (Mila 14-gauge, 13 cm, over-the-needle catheter, Mila International, Florence, KY, USA) was placed in a randomly selected jugular vein to ensure similar acepromazine administration for all horses and to provide venous access in the event of any acepromazine-associated adverse effects. After the baseline sample was collected (see below), acepromazine (Acepromazine maleate injection, 10 mg/mL, VetOne, 0.1 mg/kg bwt) was administered through the IV catheter. Administration of acepromazine in one horse was achieved by direct venipuncture in the same vein as the catheter was placed because the intravenous catheter was not patent at baseline blood collection.

The first set of samples was obtained one hour before acepromazine administration (T0), the second sample one hour after (T1), and the third sample twelve hours after acepromazine administration (T2). Samples of whole anti-coagulated (‘native’) blood for VE testing were obtained via atraumatic direct venipuncture of the non-catheterized jugular vein using an 18-gauge, 1.5-inch needle (Monoject 18-gauge Standard Hypothermic needle, Cardinal Health, Dublin, OH, USA) attached to a 3 mL syringe (Monoject 3 mL syringe, Luer-Slip Tip, Covidien). After collecting 3 mL of whole blood, an additional 40 mL of blood was obtained through the same needle in a separate syringe (Monoject, 20 mL syringe, Regular Luer Tip, Covidien) and placed into ethylenediaminetetraacetic acid (EDTA), (EDTA monoject vacutainer blood collection tubes, Covidien), sodium heparin (Heparin vacutainer blood collection tubes, Becton Dickinson, Franklin Lakes, NJ, USA), sodium citrate (Sodium Citrate plastic blood collection tubes, Fisher Scientific, Waltham, MA, USA), and plain blood collection tubes (Standard silicon-coated blood collection tubes without additives, Covidien).

Blood from the 3 mL syringe was used for VE testing within 2 min of collection using a point-of-care VE device (VCM Vet™, viscoelastic coagulation monitor, Entegrion, Durham, NC, USA) as previously described and validated for use in horses [28]. All samples were run in duplicate. The sample was gently inverted twice, and two drops of blood were discarded before placing the blood within a pre-warmed (37 °C) test cartridge. Test cartridges were then placed in the point-of-care VE testing device, and testing was initiated. VE coagulation parameters measured included clotting time (CT), clot formation time (CFT), alpha angle, maximum clot formation (MCF), amplitude at 10 min after CT (A10), amplitude at 20 min after CT (A20), lysis index at 30 min after CT (LI30), and lysis index at 45 min after CT (LI45). Several POC (point-of-care) testing devices were available, and, therefore, the devices used for each horse were randomly assigned. The same operator loaded all the cartridges, with another operator placing the cartridges into the VE test device. A separate investigator performed all venipunctures. 

Packed cell volume (PCV) and total solids (TS) were measured manually at all three time points using microhematocrit tubes (Microhematocrit tubes, plain, 75 mm, JorVet, Loveland, CO, USA) and a hand-held refractometer (Hand-held refractometer, with automatic temperature control). Additional testing was performed by the clinical pathology laboratory at the University of Illinois Veterinary Teaching Hospital. Blood samples in EDTA and sodium citrate tubes were submitted for analysis at the time of collection. Prothrombin time (PT), partial thromboplastin time (PTT), fibrinogen, and CBC were measured at all time points using automated coagulation (Compact Coagulation Analyzer, Diagnostic Stago Inc, Parsippany, NJ, USA) and hematology (Hematology analyzer, Sysmex XN 1000V, Sysmex America Inc., Lincolnshire, IL, USA) analyzers.

**Figure 1:**
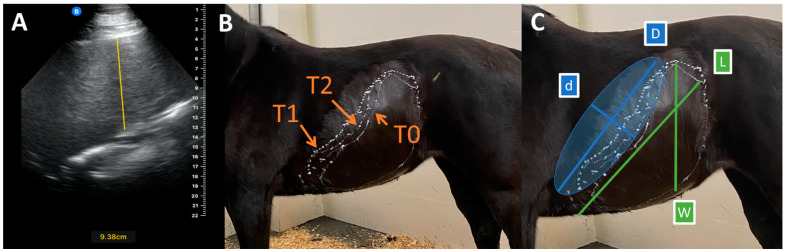
Splenic volumes were determined by ultrasonography, as described by Navas et al. [27]. (**A**) Splenic thickness measurement (vertical yellow line), showing a thickness of 9.38 cm. (**B**) Splenic shape drawn on one horse at each time point (T0 = baseline, T1 = 1 h post acepromazine, T2 = 12 h post acepromazine). (**C**) Splenic shape (drawn with white lines) and subtracted ellipsoid (faded blue shape) with measurements of width (W), length (L), width of subtracted ellipsoid (d), and length of subtracted ellipsoid (D).

Ultrasonographic measurement and calculation of splenic volume was performed at each time point following the protocol described by Navas et al. [27] using a handheld stall-side ultrasound probe (Butterfly IQ, Butterfly Network, Burlington, MA, USA). The same experienced ultrasonographer (I.M.) obtained all sonograms and measurements. The shape of the spleen at T0, T1, and T2 was marked on each horse with white correction fluid (Liquid paper, Paper mate), and measurements were performed with a tape measure (Figure 1). Measurements obtained included the following: splenic thickness (T), maximal width (W), and maximal length (L), as well as length (D) and width (d) of the subtracted ellipsoid. D was the distance from the cranial apex to the most dorsal aspect of the spleen. Twice the maximal distance from the line drawn to measure ‘D’ within the silhouette of the spleen corresponded with measurement ‘d’. Splenic thickness was measured in every intercostal space and at the flank, and mean thickness (Tmean) was calculated. The values for the ellipsoid formula were obtained by drawing an ellipsoid between the most dorsal and most cranial areas of the spleen. ‘D’ and ‘d’ were used to describe the length and width, and Tmean described the thickness. The ellipsoid for subtraction was drawn onto the horse after all other measurements were taken, and the length and width of the ellipsoid were taken. Splenic volume was calculated using both a standard ellipsoid volume formula (VOLStandard), used to estimate splenic volume in humans, and a modified formula (VOLModified), adapted to the unique shape of the equine spleen. This approach was thought to improve the accuracy due to the unique shape of the equine spleen [27].

### 2.3. Acepromazine Effect on Coagulation Ex Vivo

Five different stock solutions of acepromazine at various dilutions (1000 ng/mL; 500 ng/mL, 250 ng/mL, 125 ng/mL, and 62.5 ng/mL) were prepared with 0.9% NaCl (Sodium Chloride Injection Solution, USP, 0.9%, Medline) in serum tubes and refrigerated until use. The day before the blood draw, six 3 mL syringes were pre-loaded with 0.1 mL of each dilution and one saline control (0.9% NaCl). The pre-loaded syringes were kept refrigerated until further use. Once blood was added to the stock solutions in the syringes, the final dilutions of acepromazine in the blood were 33 ng/mL, 16.5 ng/mL, 8.25 ng/mL, 4.125 ng/mL, 2.06 mg/mL, and 0.00 ng/mL, respectively. The concentration of acepromazine in vivo was estimated at approximately 2.79 ng/mL at T1 and 0.05 ng/mL at T2 [29].

Four of the eight horses previously used were randomly selected to contribute blood for the ex vivo study. The PCV values of all horses were within reference range at the time of blood collection. Horses were restrained in stocks during venipuncture. Blood was drawn from the jugular vein via atraumatic direct venipuncture as previously described, using pre-loaded syringes. The order of the acepromazine dilutions and saline control were randomized for each horse. Each of the four horses had blood drawn at two different time points, as only three VE testing devices were available. Three different dilution samples were obtained through the same needle sequentially. VE testing was performed with VCM Vet™ immediately after obtaining blood, as described above. Jugular vein, POC testing device, and horse order were all randomly assigned. Samples for measurement of PCV, TS, CBC, fibrinogen, PT, and PTT were obtained after the last sample for VE testing was drawn, using the same needle with a 12 mL syringe (Monoject, 12 mL syringe, Regular Luer Tip, Cardinal Health). Additional testing (PCV, TS, CBC, fibrinogen, PT, and PTT) were performed as described above.

### 2.4. Statistical Analysis

Statistical analysis was performed in R version 4.2.1 using RStudio version 2022.12.0.24 [30]. Normality was confirmed using the Shapiro–Wilk test and visual assessment of the data. Summary data were represented as the median and interquartile range (IQR). For the in vivo study, one-way repeated measures ANOVA was performed to determine the effect of time (sample) on VE coagulation and CBC parameters as well as splenic volume. Where a significant effect was identified, pairwise comparisons between groups were performed by Tukey’s HSD test. Residuals were evaluated to confirm that assumptions of normality and homogeneity of variance were met for all data analyzed. *P*-values ≤ 0.05 were considered significant. For the in vitro study, two-way repeated measures ANOVA was used to determine the effect of horse and acepromazine concentration on viscoelastic coagulation parameters. Figures were created with ggpubr package version 0.6.0 [31]; the packages kableExtra version 1.3.4 [32] and magicfor version 0.1.0 [33] were used for processing and visualization of the data.

## 3. Results

All horses were considered normal on physical examination prior to project start. CBC, blood chemistry, and fibrinogen prior to enrollment showed no clinically significant abnormalities.

**Figure 2:**
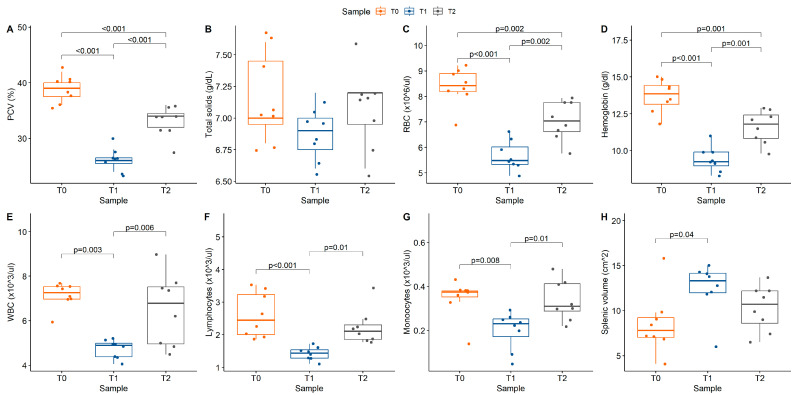
Box plots with overlaid scatter illustrating hematologic parameters of interest: (**A**) packed cell volume (PCV), *p* < 0.001; (**B**) total solids (TS), *p* = 0.15; (**C**) red blood cell count (RBC), *p* < 0.001; (**D**) hemoglobin, *p* < 0.001; (**E**) white blood cell count (WBC), *p* = < 0.001; (**F**) lymphocytes, *p* < 0.001; (**G**) monocytes, *p* = 0.005; (**H**) modified splenic volume, *p* = 0.045. Color reflects study time points (T0 = baseline, T1 = 1 h post acepromazine, T2 = 12 h post acepromazine). Values above brackets reflect significance of between-group comparisons.

Baseline PCV ranged from 36% to 42% in the horses prior to acepromazine administration. Acepromazine administration successfully achieved a decrease in PCV to below the reference range in all the horses one hour after administration (approximately 13% points from baseline; T0–T1 *p* < 0.001; Table 1, Figure 2). PCV increased from T1 to T2 (*p* = 0.002) but remained lower at T2 (range 28–36%) than at T0 (*p* = 0.002). Red cell parameters on hematology (red blood cell count, hemoglobin, hematocrit) were consistent with these findings and also decreased after acepromazine administration (Table 1). White blood cell count (WBC) significantly decreased from T0 (7.26 × 10^3^ cells/μL [6.97,7.54]) to T1 (4.89 × 10^3^ cells/μL [4.38,4.99]; *p* < 0.001) before returning to baseline at T2 (6.78 × 10^3^ cells/μL [4.65,7.52]. The change in WBC was attributed to corresponding changes in absolute lymphocyte (*p* < 0.001) and monocyte (*p* = 0.005) counts. Splenic volume was significantly increased at T1, using both standard and modified formulas, and returned to baseline at T2 (Table 2).

**Figure 3:**
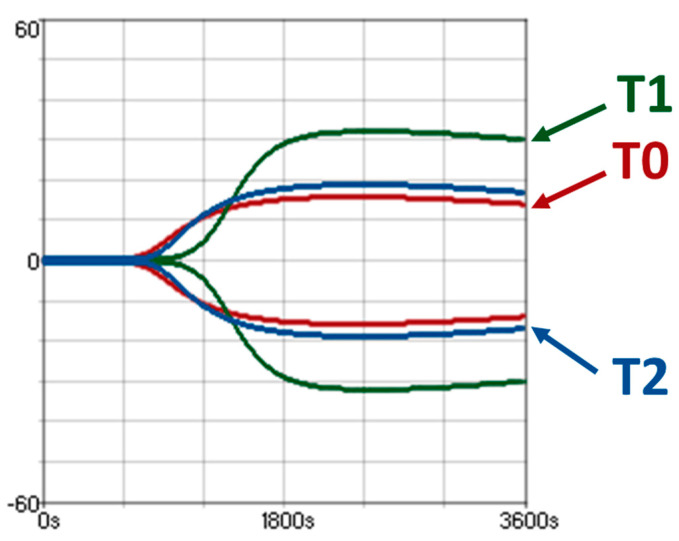
Viscoelastic coagulation profiles on a VCM Vet^TM^ device from horse 7 at all three times points as overlapping viscoelastic traces (T0 = baseline, red trace; T1 = 1 h post acepromazine, green trace; T2 = 12 h post acepromazine, blue trace).

**Figure 4:**
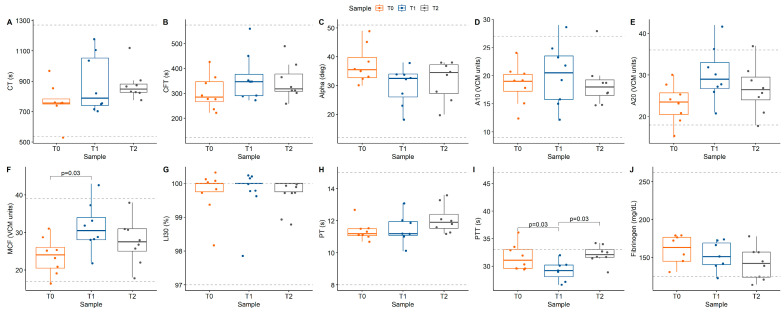
Box plots with overlaid scatter illustrating viscoelastic and plasma-based coagulation parameters: (**A**) CT (*p* = 0.21), (**B**) CFT (*p* = 0.33), (**C**) alpha angle (*p* = 0.097), (**D**) A10 (*p* = 0.75), (**E**) A20 (*p* = 0.068), (**F**) MCF (*p* = 0.044) (**G**) LI30 (*p* = 0.91), (**H**) PT (*p* = 0.178), (**I**) PTT (*p* = 0.024), and (**J**) fibrinogen (*p* = 0.213). Color reflects study time points (T0 = baseline, T1 = 1 h post acepromazine, T2 = 12 h post acepromazine). Dashed lines represent upper and lower limits of reference range. Values above brackets reflect significance of between-group comparisons.

**Figure 5:**
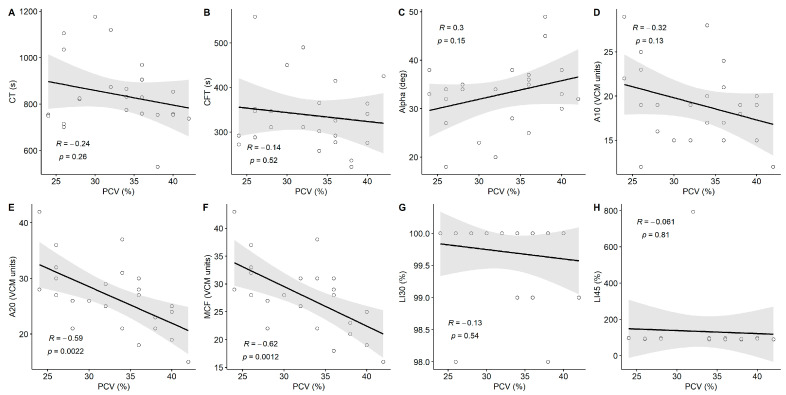
Scatter plot of viscoelastic coagulation parameters vs. packed cell volume (PCV) over the entire study. (**A**) CT, (**B**) CFT, (**C**) alpha, (**D**) A10, (**E**) A20, (**F**) MCF, (**G**) LI30, and (**H**) LI45. Lines and gray shaded area represent results of Pearson’s correlation analysis with 95% confidence intervals, respectively.

There was evidence of hypercoagulability on both VE and plasma-based coagulation testing (Figure 3; Table 3). MCF increased from T0 (24 VCM units [20.5,26]) to T1 (30.5 VCM units [28,34]; *p* = 0.03). PTT decreased from T0 (31.1 s [29.6,33]) to T1 (29.2 s [28.1,30.2]) before increasing at T2 (32.1 s [31.6,33]; *p* = 0.03). All other coagulation parameters remained within reference ranges (Figure 4). There was a significant linear correlation between PCV and A20 (R = −0.59, *p* = 0.002) and MCF (R = −0.62, *p* = 0.001), with apparent hypercoagulability occurring at a lower PCV (Figure 5).

In the ex vivo portion of the study, all the horses had normal VE and plasma-based coagulation tests at baseline. There was a significant effect of horse on CT (*p* < 0.001), CFT (*p* = 0.02), and alpha (*p* < 0.001), but there was no significant effect of acepromazine concentration on any VE coagulation parameter (Table 4).

## 4. Discussion

The findings of this study demonstrate that acepromazine-induced splenic relaxation in healthy adult horses created a transient decrease in red cell mass, substantiating the use of this model to create normovolemic non-inflammatory acute anemia. Decreased RCM resulted in hypercoaguability, as identified by both VE and plasma-based coagulation testing, indicated by increased MCF and decreased PTT. The ex vivo addition of acepromazine to native whole blood had no significant effect on VE coagulation parameters. While the effect of polycythemia (increased RCM) on VE testing in horses has been well documented [18,20], to our knowledge, this is the first report of decreased RCM on VE testing in horses. Understanding the dynamic relationship between RCM and VE coagulation testing is essential for clinical interpretation.

Decreases in RCM have been previously associated with altered VE coagulation parameters in human patients suffering from hemolytic anemia [34], as well as surgical blood loss and subsequent hemodilution [35]. In dogs, a whole-blood removal followed by volume restoration with crystalloid fluids model resulted in apparent ROTEM hypercoagulability, shown as changes in CT, CFT, alpha angle, and MCF [19].

Conversely, two studies using a phenylephrine-induced polycythemia model in healthy horses revealed VE hypocoagulability [18,20]. Neither study identified clinical signs of coagulopathy, and plasma-based coagulation tests remained within normal limits, suggesting that the changes in RCM and associated viscoelastic coagulation parameters could be artefactual. Polycythemia, either physiologic or experimental, has been associated with coagulation testing abnormalities in humans and other animals [36,37,38]. The theoretical mechanistic explanation is that an increase in red blood cells decreases the available plasma volume, and therefore plasma coagulation factors, inside the contained testing devices, resulting in artifactual hypocoagulability. Anemia might therefore have the opposite effect and cause an increased concentration of coagulation factors in plasma within the testing cup, resulting in hypercoagulable viscoelastic testing results [18,19].

The current study also showed no clinical or laboratory-based hypocoagulability associated with induced anemia. Instead, there was laboratory evidence of hypercoagulability (increased MCF and decreased PTT), contradicting findings from human patients with clinical illness. For example, anemia was associated with a higher risk of major bleeding in patients suffering from acute coronary syndrome [39]. In patients with atrial fibrillation, anemia has been associated with bleeding tendency but not stroke risk or thromboembolic events [40].

There are limited studies demonstrating hypercoagulability with plasma-based coagulation testing associated with anemia. In the past, a shortened PTT was mainly attributed to pre-analytic errors such as flawed blood collection, sample handling, or sample storage [41]. More recent studies, however, revealed that human patients with shortened aPTT are more likely to develop venous thromboembolism [42]. Moreover, women suffering from anemia due to adenomyosis showed possible anemia-associated hypercoagulability, as indicated by an increased platelet count and PT. It remains unclear if these changes are solely attributed to anemia or are part of underlying inflammatory processes [43].

It is possible that the changes found in our study indicate true hypercoagulability. However, no clinical signs of coagulopathy, such as thrombus formation associated with the catheter insertion or venipuncture sites, were observed in any of the horses during the 24 h after acepromazine administration.

Although not statistically significant, a tendency towards decreased total protein (TP) was noted following acepromazine administration. A similar tendency was noted with fibrinogen, which also decreased at T1 and T2, likely reflecting total protein changes, as fibrinogen makes a portion of the plasma protein and, therefore, total protein. Considering that acepromazine can cause hypotension, a decrease in TS may be associated with increased plasma volume secondary to that hypotension [21]. The fact that the TS decrease was less notable in the current study and was not statistically significant could be explained by a relatively minor change in plasma volume compared to the impressive storage capacity for red blood cells in the spleen. This deserves future examination, as the plasma volume was not determined in the current study.

CBC changes in this study included alterations in white blood cell count and differential parameters, as well as red blood cell parameters. RBC, hemoglobin, and hematocrit decreased after acepromazine administration, in parallel with PCV, as anticipated, and there was a concurrent decrease in white blood cell count, attributed to decreases in absolute lymphocyte and monocyte counts. These findings are consistent with previous reports stating that both white and red cell parameters decrease after administration of sedatives, including acepromazine [22,24]. The changes mentioned above might be attributed to the sequestration of white blood cells in the spleen following splenic capsule relaxation, similar to the observed decrease in PCV. The decreased white blood cell count could also be attributed to increase in plasma volume secondary to hypotension, as described for TS above.

Several limitations were associated with this study, including the fact that only a small number of horses were investigated. Anemia was induced in healthy horses, which may not be fully representative of changes associated with clinical illness or blood loss. Further evaluation of the relationship between PCV and VE coagulation in clinical patients is warranted. A distinction between true hypercoagulability and artifactual changes could not be made with certainty in the current work. In order to confirm functional hypercoagulability, template bleeding time (TBT) could be used as an additional assessment. However, TBT has been shown to have poor reproducibility and a high inter-individual range in horses, making it of limited use in the diagnosis of coagulopathy in horses [44]. The ultrasonographer was not blinded to the time point of acepromazine administration, and they therefore might have been biased while evaluating splenic size. Additionally, in this study, each horse was allowed as its own control, so an additional saline control group was not used. A saline control group sampled at similar times could have provided additional information.

## 5. Conclusions

In conclusion, decreased RCM in horses resulted in apparent hypercoagulability based on VE and plasma-based coagulation testing. These changes did not result in clinically apparent coagulopathy in healthy horses and could represent an artifact. Considering these findings in conjunction with results from past equine studies examining RCM changes and viscoelastic testing, care must be taken when interpreting viscoelastic coagulation profiles in equine patients presenting with abnormal PCV values. Further studies are needed to develop appropriate reference ranges for VE coagulation profiles in anemic and polycythemic equine patients.

## Figures and Tables

**Table 1:** Stall-side PCV/TS and hematology parameters for each time point (T0 = baseline prior to acepromazine administration, T1 = 1 h after acepromazine administration, T2 = 12 h after acepromazine administration) presented as median (Q1, Q3). *p*-values reflect results from one-way repeated measures ANOVA, * denotes statistical significance. Where applicable, values within a row that have different superscripts (^A^, ^B^, ^C^) were significantly different on Tukey’s HSD post hoc test.

	REF	T0	T1	T2	*p*-Value
PCV (%)	32–42	39 (37.5, 40) ^A^	26 (25.5, 26.5) ^B^	34 (32, 34.5) ^C^	<0.001 *
TS (g/dL)	5.5–7.3	7 (6.95, 7.95)	6.9 (6.75, 7)	7.2 (6.95, 7.2)	0.15
RBC (×10^6^ cells/μL)	7.00–13.0	8.46 (8.19, 8.91) ^A^	5.48 (5.33, 6.02) ^B^	7.06 (6.61, 7.76) ^C^	<0.001 *
Hemoglobin (g/dL)	11.0–19.0	13.85 (13.15, 14.43) ^A^	9.25 (8.98, 9.9) ^B^	11.8 (10.83, 12.43) ^C^	<0.001 *
Hematocrit (%)	32.0–53.0	39.56 (38.4, 41.25) ^A^	27 (29.93, 28.43) ^B^	34.4 (31.98, 36.5) ^C^	<0.001 *
MCV (fL)	32.0–53.0	47.55 (46.7, 48.43)	48.2 (46.7, 49.08)	48.35 (47.5, 49.33)	0.79
MCH (pg)	12.0–20.0	16.5 (15.58, 16.75)	16.8 (16.38, 16.93)	16.7 (16.25, 16.93)	0.632
MCHC (g/dL)	30.0–39.0	34.55 (34.2, 34.88)	34.75 (33.9, 34.98)	34.20 (33.9, 34.55)	0.468
Platelets (×10^3^ cells/μL)	100–600	143.50 (135.5, 152.5)	150.50 (137.25, 164.75)	139.00 (131.25, 173)	0.975
WBC (×10^3^ cells/μL)	5.50–12.0	7.26 (6.97, 7.54) ^A^	4.89 (4.38, 4.99) ^B^	6.78 (4.95, 7.52) ^A^	<0.001 *
Neutrophils (×10^3^ cells/μL)	3.00–7.00	3.84 (3.4, 4.56)	3.12 (2.7, 3.44)	3.81 (2.56, 4.32)	0.112
Lymphocytes (×10^3^ cells/μL)	1.50–5.00	2.45 (2.01, 3.23) ^A^	1.44 (1.29, 1.54) ^B^	2.11 (1.86, 2.3) ^A^	<0.001 *
Monocytes (×10^3^ cells/μL)	0.00–1.00	0.38 (0.35, 0.38) ^A^	0.23 (0.17, 0.25) ^B^	0.31 (0.29, 0.41) ^A^	0.005 *
Eosinophils (×10^3^ cells/μL)	0.00–1.00	0.13 (0.13, 0.22)	0.08 (0.05, 0.01)	0.11 (0.08, 0.15)	0.262
Basophils (×10^3^ cells/μL)	0.00–2.00	0.03 (0.03, 0.05)	0.02 (0.01, 0.02)	0.03 (0.02, 0.04)	0.123

Abbreviations: MCH, mean corpuscular hemoglobin; MCHC, mean corpuscular hemoglobin concentration; MCV, mean corpuscular volume; PCV, packed cell volume; RBC, red blood cell count; REF, institutional reference range; TS, total solids; WBC, white blood cells.

**Table 2:** Splenic ultrasound measurements and splenic volume calculation with both standard (VOLStandard) and modified (VOLModified) formulas, as previously published by Navas et al. [27], for each time point (T0 = baseline prior to acepromazine administration, T1 = 1 h after acepromazine administration, T2 = 12 h after acepromazine administration) presented as median (Q1, Q3). *p*-values reflect results from one-way repeated measures ANOVA, * denotes statistical significance. Where applicable, values within a row that have different superscripts (^a^, ^b^) were significantly different on Tukey’s HSD post hoc test.

	T0	T1	T2	*p*-Value
VOLStandard (L)	11.3 (9.725, 12.425) ^a^	16.9 (14.925, 17.875) ^b^	15.150 (12.425, 16.425) ^a,b^	0.032 *
VOLModified (L)	7.8 (7.025, 9.2) ^a^	13.3 (11.98, 14.15) ^b^	10.7 (8.6, 12.2 ^a,b^	0.045 *

**Table 3:** Values of coagulation parameters, including viscoelastic testing (VCM Vet^TM^), plasma-based coagulation, and platelet count for each time point (T0 = baseline, T1 = 1 h post acepromazine, T2 = 12 h post acepromazine), presented as median (Q1, Q3). *p*-values reflect results from one-way repeated measures ANOVA, * denotes statistical significance. Where applicable, values within a row that have different superscripts (^a^, ^b^) were significantly different on Tukey’s HSD post hoc test.

	REF	T0	T1	T2	*p*-Value
CT (s)	536–1270	756 (750, 783)	788 (741, 1053)	849 (828, 882)	0.21
CFT (s)	123–574	284 (266, 347)	347 (291, 377)	318.5 (309, 378)	0.33
Alpha (deg)	12–51	35.5 (33, 40)	32.5 (26, 34)	34.5 (27, 37)	0.097
A10 (VCM units)	9–27	19 (17, 20)	20.5 (16, 24)	18 (17, 19)	0.75
A20 (VCM units)	18–36	24 (21, 26)	29 (27, 33)	27 (24, 30)	0.068
MCF (VCM units)	17–39	24 (21, 26) ^a^	31 (28, 34) ^b^	28 (25, 31) ^a,b^	0.044 *
LI30 (%)	97–100	100 (100, 100)	100 (100, 100)	100 (100, 100)	0.91
LI45 (%)	82–100	95 (91, 96)	96 (94, 96)	95 (91, 96)	0.399
Fibrinogen (mg/dL)	103–254	163 (145, 177)	151 (141, 169)	142 (124, 157)	0.213
PT (s)	8–15	11 (11, 12)	11 (11, 12)	12 (12, 12)	0.178
PTT (s)	33–47	31 (30, 33) ^a^	30 (28, 30) ^b^	32 (32, 33) ^a^	0.024 *
Platelets (×10^3^ cells/μL)	100–600	144 (136, 153)	151 (137, 165)	139 (131, 173)	0.975

Abbreviations: CFT, clot formation time; CT, clot time; LI, clot lysis; MCF, mean clot formation; PT, prothrombin time; PTT, activated partial thromboplastin time; REF, institutional reference range [28].

**Table 4:** Viscoelastic coagulation parameters from ex vivo study, in which five concentrations of acepromazine were added to native whole blood during collection. *p*-values indicate results from two-way repeated measures ANOVA for effects of concentration (CONC) and horse; * denotes statistical significance.

	1000 ng/mL	500 ng/mL	250 ng/mL	125 ng/mL	62.5 ng/mL	0 ng/mL	Conc	Horse
CT (s)	974 (884, 1056)	900 (849, 910)	850 (837, 982)	877 (790, 984)	809 (806, 886)	908 (778, 977)	0.207	<0.001 *
CFT (s)	381 (350, 428)	287 (234, 326)	351 (318, 425)	380 (317, 447)	363 (319, 410)	276 (212, 437)	0.972	0.023 *
Alpha (deg)	31.5 (26, 37)	35 (32, 39)	36 (31, 37)	31 (29, 34)	31 (30, 34)	40 (29, 48)	0.3	<0.001 *
A10 (VCM units)	14.5 (13, 16)	19 (18, 20)	14 (13, 17)	14 (13 17)	17 (15, 19)	22 (17, 24)	0.452	0.158
A20 (VCM units)	20 (18, 22)	25 (24, 27)	18 (18, 22)	18 (17, 21)	22 (20, 24)	28 (24, 28)	0.654	0.094
MCF (VCM units)	20 (19, 24)	25 (24, 27)	18 (18, 22)	19 (17, 22)	23 (21, 24)	28 (24, 28)	0.891	0.297
LI30 (%)	100 (94, 100)	100 (99, 100)	100 (100, 100)	100 (100, 100)	100 (100, 100)	100 (100, 100)	0.063	0.329
LI45 (%)	94 (94, 94)	93 (93, 93)	94 (94, 94)	95 (94, 96)	94 (93, 97)	92 (89, 94)	1	0.238

Abbreviations: CFT, clot formation time; CT, clot time; LI, clot lysis; MCF, maximum clot formation.

## Data Availability

Mersich, Ina; Bishop, Rebecca; Diaz Yucupicio, Sandra; Nobrega, Ana D.; Austin, Scott; Barger, Anne; Fick, Megan E.; Wilkins, Pamela (2024): Data for Decreased Circulating Red Cell Mass (Packed Cell Volume) Alters Viscoelastic and Traditional Plasma Coagulation Testing Results in Healthy Horses. University of Illinois at Urbana-Champaign. Data from this study is available at: https://doi.org/10.13012/B2IDB-9153919_V1.
